# CAR-T “the living drugs”, immune checkpoint inhibitors, and precision medicine: a new era of cancer therapy

**DOI:** 10.1186/s13045-019-0819-1

**Published:** 2019-11-08

**Authors:** Delong Liu

**Affiliations:** 1 New York Medical College, Valhalla, NY 10595 USA; 2 The First Affiliated Hospital of Zhengzhou University, Zhengzhou, 450052 China

**Keywords:** Cancer immunotherapy, CAR-T, TCR-T, Immune checkpoint inhibitor

## Abstract

New advances in the design and manufacture of monoclonal antibodies, bispecific T cell engagers, and antibody-drug conjugates make the antibody-directed agents more powerful with less toxicities. Small molecule inhibitors are routinely used now as oral targeted agents for multiple cancers. The discoveries of PD1 and PD-L1 as negative immune checkpoints for T cells have led to the revolution of modern cancer immunotherapy. Multiple agents targeting PD1, PD-L1, or CTLA-4 are widely applied as immune checkpoint inhibitors (ICIs) which alleviate the suppression of immune regulatory machineries and lead to immunoablation of once highly refractory cancers such as stage IV lung cancer. Tisagenlecleucel and axicabtagene ciloleucel are the two approved CD19-targeted chimeric antigen receptor (CAR) T cell products. Several CAR-T cell platforms targeting B cell maturation antigen (BCMA) are under active clinical trials for refractory and/or relapsed multiple myeloma. Still more targets such as CLL-1, EGFR, NKG2D and mesothelin are being directed in CAR-T cell trials for leukemia and solid tumors. Increasing numbers of novel agents are being studied to target cancer-intrinsic oncogenic pathways as well as immune checkpoints. One such an example is targeting CD47 on macrophages which represents a “do-not-eat-me” immune checkpoint. Fueling the current excitement of cancer medicine includes also TCR- T cells, TCR-like antibodies, cancer vaccines and oncolytic viruses.

Monoclonal antibodies (MoAb) targeting CD20 with rituximab, ofatumumab, and obinutumumab have led to a paradigm shift in B cell lymphoma and leukemia therapy [1, 2]. MoAbs targeting HER2 are widely used for breast cancer therapy [3, 4]. Small molecular inhibitors such as tyrosine kinase inhibitors (TKI) have become a major modality of therapy for a variety of cancers [5, 6]. The recent approval of chimeric antigen receptor (CAR) – engineered T cells targeting CD19 has opened a new era with “living drugs” for cancer immunotherapy [7–9]. The two collections of “Emerging agents and regimens for cancer therapy” and “Cancer immunotherapy: recent advances and future perspectives” summarized latest development in the therapy for different cancer types and the search for novel targets of cancer immunotherapy. Major advances in the following fields are particularly encouraging and promising.

## Antibodies: more on-target and less off-tumor effects

New advances in the design and manufacture of MoAbs, Bispecific T cell engagers (BiTEs), and antibody-drug conjugates (ADCs) make the antibody- directed agents more powerful with less toxicities [1, 10–12]. Blinatumomab as the first approved CD19-targeted BiTE is being studied for induction therapy for elderly patients with acute lymphoblastic leukemia (ALL) and for incorporation into the regimens containing the CD22-targeted ADC, inotuzumab ozogamicin, in an attempt to enhance efficacy and reduce toxicities [13–15]. ADCs targeting CD30, CD33, or CD79 have been approved for clinical therapy of lymphomas and AML with the appropriate targets [16–18]. BiTEs for solid tumors are under active clinical trials [19, 20].

## Small molecule inhibitors (SMI) as targeted agents: small pills, big impact

Imatinib opened a new era of targeted therapies with oral SMIs [21]. BCR-ABL tyrosine kinase inhibitors (TKI) have fundamentally changed the therapeutic paradigm of chronic myeloid leukemia (CML) and possibly of ALL with BCR-ABL mutations in the near future [22, 23]. JAK2 inhibitors, ruxolitinib and fedratinib, are major therapy options for myelofibrosis [24–26]. Inhibitors for BCL-2, venetoclax, and Bruton tyrosine kinase, ibrutinib and acalabrutinib, are playing major roles in therapy for chronic lymphoid leukemia as well as in mantle cell lymphoma [27–30]. Recently, FLT3 inhibitors and inhibitors of isocitrate dehydrogenases (IDH1 and IDH2) significantly enhanced the armamentarium for AML therapy [31–35]. TKIs targeting a variety of oncoproteins, such as EGFR, ALK, HER2, FGFR, VEGFR, RET, MET, to name a few, have brought revolutions in the therapy of non-small cell lung cancer, breast cancer, bladder cancer, liver cancer, and renal cell carcinoma [5, 6, 36–42]. BRAF inhibitors targeting serine /threonine kinases lead to major advances in the therapy of malignant melanoma [43, 44]. PARP inhibitors and CDK inhibitors significantly expanded the weaponry for breast and ovarian cancers [45–50].

## Immune checkpoint inhibitors (ICI): targeting tumor microenvironment, restoring immune function

The discoveries of PD1 and PD-L1 have led to the revolution of modern cancer immunotherapy [51]. Multiple agents targeting PD1, PD-L1, or CTLA-4 either as single agent or combination regimens are widely used as ICIs which alleviate the suppression of immune regulatory machineries and lead to immunoablation of once highly refractory cancer cells [52–55]. Recent discoveries on the immunomodulatory effects of gut microbiota shed lights on new ways in enhancing cancer immunotherapy [56].

## CAR-T cells: living drugs

Tisagenlecleucel, the first approved CD19-targeted CAR-T cells, have been in clinical applications for refractory /relapsed (RR) ALL and large B cell lymphoma (LBCL) [8, 9, 57]. Axicabtagene ciloleucel is also approved for LBCL [9]. Several CAR-T cell products targeting B cell maturation antigen (BCMA) as well as CD19 are under active clinical trials for RR multiple myeloma [58–60]. Several biomarkers such as CLL-1, EGFR, NKG2D, and mesothelin are being targeted in CAR-T cell trials for leukemia and solid tumors [61–66]. Dual-target CAR-T cells and sequential or cocktail CAR-T cell trials have been shown to provide clinical benefits for highly refractory cancers [67]. Universal CARs are being engineered and universal CAR-T cells are in clinical trials [68, 69]. Recent discoveries in mechanisms for CAR-T toxicities (CARTox), such as cytokine release syndrome and neurotoxicities, suggest that prophylaxis for CARTox may not affect efficacy of CAR-T cells [70, 71]. These discoveries make it possible to preemptively or prophylactically treat and minimize CARTox [72–74].

## Novel agents targeting new signaling pathways, biomarkers, and immune checkpoints

mTOR inhibitors, such as everolimus and temsirolimus, target and block a significant signaling pathway that proves vital for PI3K/AKT signal transduction [75]. New inhibitors for inflammasomes are being studied [76]. These novel inhibitors represent new families of targeted agents. Recently, tumor-associated macrophages in the tumor microenvironment are increasingly recognized to facilitate cancer metastasis [77]. One active approach in early clinical trials is targeting CD47 on the macrophage cell surface that represents a “do-not-eat-me” immune checkpoint molecule [78, 79]. TCR- T cells, TCR-like antibodies, cancer vaccines and oncolytic viruses are fueling new endeavors for cancer immunotherapy [80–83]. The CAR-T “living drugs”, small molecule inhibitors, and immune checkpoint inhibitors mark a new era of cancer therapy.

## Data Availability

This is not applicable.

## Abbreviation

CAR: Chimeric antigen receptor

## References

[1] Goede V, Fischer K, Busch R, Engelke A, Eichhorst B, Wendtner CM, Chagorova T, de la Serna J, Dilhuydy MS, Illmer T, Opat S, Owen CJ, Samoylova O, Kreuzer KA, Stilgenbauer S, Dohner H, Langerak AW, Ritgen M, Kneba M, Asikanius E, Humphrey K, Wenger M, Hallek M (2014). Obinutuzumab plus chlorambucil in patients with CLL and coexisting conditions. N Engl J Med.

[2] Maury Sébastien, Chevret Sylvie, Thomas Xavier, Heim Dominik, Leguay Thibaut, Huguet Françoise, Chevallier Patrice, Hunault Mathilde, Boissel Nicolas, Escoffre-Barbe Martine, Hess Urs, Vey Norbert, Pignon Jean-Michel, Braun Thorsten, Marolleau Jean-Pierre, Cahn Jean-Yves, Chalandon Yves, Lhéritier Véronique, Beldjord Kheira, Béné Marie C., Ifrah Norbert, Dombret Hervé (2016). Rituximab in B-Lineage Adult Acute Lymphoblastic Leukemia. New England Journal of Medicine.

[3] Slamon D, Eiermann W, Robert N, Pienkowski T, Martin M, Press M, Mackey J, Glaspy J, Chan A, Pawlicki M, Pinter T, Valero V, Liu M-C, Sauter G, von Minckwitz G, Visco F, Bee V, Buyse M, Bendahmane B, Tabah-Fisch I, Lindsay M-A, Riva A, Crown J (2011). Adjuvant Trastuzumab in HER2-positive breast Cancer. N Engl J Med.

[4] Verma S, Miles D, Gianni L, Krop IE, Welslau M, Baselga J, Pegram M, Oh D-Y, Diéras V, Guardino E, Fang L, Lu MW, Olsen S, Blackwell K (2012). Trastuzumab Emtansine for HER2-positive advanced breast Cancer. N Engl J Med.

[5] Mok TS, Wu Y-L, Ahn M-J, Garassino MC, Kim HR, Ramalingam SS, Shepherd FA, He Y, Akamatsu H, Theelen WSME, Lee CK, Sebastian M, Templeton A, Mann H, Marotti M, Ghiorghiu S, Papadimitrakopoulou VA (2016). Osimertinib or platinum–Pemetrexed in EGFR T790M–positive lung Cancer. N Engl J Med.

[6] Shaw AT, Kim D-W, Nakagawa K, Seto T, Crinó L, Ahn M-J, De Pas T, Besse B, Solomon BJ, Blackhall F, Wu Y-L, Thomas M, O'Byrne KJ, Moro-Sibilot D, Camidge DR, Mok T, Hirsh V, Riely GJ, Iyer S, Tassell V, Polli A, Wilner KD, Jänne PA (2013). Crizotinib versus chemotherapy in advanced ALK-positive lung Cancer. N Engl J Med.

[7] Garfall AL, Maus MV, Hwang WT, Lacey SF, Mahnke YD, Melenhorst JJ, Zheng Z, Vogl DT, Cohen AD, Weiss BM, Dengel K, Kerr ND, Bagg A, Levine BL, June CH, Stadtmauer EA (2015). Chimeric antigen receptor T cells against CD19 for multiple myeloma. N Engl J Med.

[8] Maude SL, Laetsch TW, Buechner J, Rives S, Boyer M, Bittencourt H, Bader P, Verneris MR, Stefanski HE, Myers GD, Qayed M, De Moerloose B, Hiramatsu H, Schlis K, Davis KL, Martin PL, Nemecek ER, Yanik GA, Peters C, Baruchel A, Boissel N, Mechinaud F, Balduzzi A, Krueger J, June CH, Levine BL, Wood P, Taran T, Leung M, Mueller KT (2018). Tisagenlecleucel in children and Young adults with B-cell lymphoblastic leukemia. N Engl J Med.

[9] Neelapu SS, Locke FL, Bartlett NL, Lekakis LJ, Miklos DB, Jacobson CA, Braunschweig I, Oluwole OO, Siddiqi T, Lin Y, Timmerman JM, Stiff PJ, Friedberg JW, Flinn IW, Goy A, Hill BT, Smith MR, Deol A, Farooq U, McSweeney P, Munoz J, Avivi I, Castro JE, Westin JR, Chavez JC, Ghobadi A, Komanduri KV, Levy R, Jacobsen ED, Witzig TE (2017). Axicabtagene Ciloleucel CAR T-cell therapy in refractory large B-cell lymphoma. N Engl J Med.

[10] Kantarjian H, Stein A, Gokbuget N, Fielding AK, Schuh AC, Ribera JM, Wei A, Dombret H, Foa R, Bassan R, Arslan O, Sanz MA, Bergeron J, Demirkan F, Lech-Maranda E, Rambaldi A, Thomas X, Horst HA, Bruggemann M, Klapper W, Wood BL, Fleishman A, Nagorsen D, Holland C, Zimmerman Z, Topp MS (2017). Blinatumomab versus chemotherapy for advanced acute lymphoblastic leukemia. N Engl J Med.

[11] Kantarjian HM, DeAngelo DJ, Stelljes M, Martinelli G, Liedtke M, Stock W, Gokbuget N, O'Brien S, Wang K, Wang T, Paccagnella ML, Sleight B, Vandendries E, Advani AS (2016). Inotuzumab Ozogamicin versus standard therapy for acute lymphoblastic leukemia. N Engl J Med.

[12] Topp MS, Gökbuget N, Stein AS, Zugmaier G, O'Brien S, Bargou RC, Dombret H, Fielding AK, Heffner L, Larson RA, Neumann S, Foà R, Litzow M, Ribera J-M, Rambaldi A, Schiller G, Brüggemann M, Horst HA, Holland C, Jia C, Maniar T, Huber B, Nagorsen D, Forman SJ, Kantarjian HM (2015). Safety and activity of blinatumomab for adult patients with relapsed or refractory B-precursor acute lymphoblastic leukaemia: a multicentre, single-arm, phase 2 study. The Lancet Oncology.

[13] Aujla A, Aujla R, Liu D (2019). Inotuzumab ozogamicin in clinical development for acute lymphoblastic leukemia and non-Hodgkin lymphoma. Biomarker Research.

[14] Liu D, Zhao J, Song Y, Luo X, Yang T (2019). Clinical trial update on bispecific antibodies, antibody-drug conjugates, and antibody-containing regimens for acute lymphoblastic leukemia. J Hematol Oncol.

[15] Yu B, Liu D (2019). Antibody-drug conjugates in clinical trials for lymphoid malignancies and multiple myeloma. J Hematol Oncol.

[16] Stanchina M, Pastore A, Devlin S, Famulare C, Stein E, Taylor J (2019). CD33 splice site genotype was not associated with outcomes of patients receiving the anti-CD33 drug conjugate SGN-CD33A. J Hematol Oncol.

[17] Morschhauser F, Flinn IW, Advani R, Sehn LH, Diefenbach C, Kolibaba K, Press OW, Salles G, Tilly H, Chen AI, Assouline S, Cheson BD, Dreyling M, Hagenbeek A, Zinzani PL, Jones S, Cheng J, Lu D, Penuel E, Hirata J, Wenger M, Chu YW, Sharman J (2019). Polatuzumab vedotin or pinatuzumab vedotin plus rituximab in patients with relapsed or refractory non-Hodgkin lymphoma: final results from a phase 2 randomised study (ROMULUS). Lancet Haematol.

[18] Younes A, Bartlett NL, Leonard JP, Kennedy DA, Lynch CM, Sievers EL, Forero-Torres A (2010). Brentuximab vedotin (SGN-35) for relapsed CD30-positive lymphomas. N Engl J Med.

[19] Benonisson H, Altıntaş I, Sluijter M, Verploegen S, Labrijn AF, Schuurhuis DH, Houtkamp MA, Verbeek JS, Schuurman J, van Hall T (2019). CD3-Bispecific antibody therapy turns solid tumors into inflammatory sites but does not install protective memory. Mol Cancer Ther.

[20] Cully M (2017). Bispecific antibody directs T cells to solid tumours. Nat Rev Drug Discov.

[21] Hochhaus A, Larson RA, Guilhot F, Radich JP, Branford S, Hughes TP, Baccarani M, Deininger MW, Cervantes F, Fujihara S, Ortmann C-E, Menssen HD, Kantarjian H, O’Brien SG, Druker BJ (2017). Long-term outcomes of Imatinib treatment for chronic myeloid leukemia. N Engl J Med.

[22] Kantarjian H, Shah NP, Hochhaus A, Cortes J, Shah S, Ayala M, Moiraghi B, Shen Z, Mayer J, Pasquini R, Nakamae H, Huguet F, Boqué C, Chuah C, Bleickardt E, Bradley-Garelik MB, Zhu C, Szatrowski T, Shapiro D, Baccarani M (2010). Dasatinib versus Imatinib in newly diagnosed chronic-phase chronic myeloid leukemia. N Engl J Med.

[23] Saglio G, Kim D-W, Issaragrisil S, le Coutre P, Etienne G, Lobo C, Pasquini R, Clark RE, Hochhaus A, Hughes TP, Gallagher N, Hoenekopp A, Dong M, Haque A, Larson RA, Kantarjian HM (2010). Nilotinib versus Imatinib for newly diagnosed chronic myeloid leukemia. N Engl J Med.

[24] Harrison CN, Schaap N, Vannucchi AM, Kiladjian JJ, Tiu RV, Zachee P, Jourdan E, Winton E, Silver RT, Schouten HC, Passamonti F, Zweegman S, Talpaz M, Lager J, Shun Z, Mesa RA (2017). Janus kinase-2 inhibitor fedratinib in patients with myelofibrosis previously treated with ruxolitinib (JAKARTA-2): a single-arm, open-label, non-randomised, phase 2, multicentre study. Lancet Haematol.

[25] Kvasnicka HM, Thiele J, Bueso-Ramos CE, Sun W, Cortes J, Kantarjian HM, Verstovsek S (2018). Long-term effects of ruxolitinib versus best available therapy on bone marrow fibrosis in patients with myelofibrosis. J Hematol Oncol.

[26] Li B, Rampal RK, Xiao Z (2019). Targeted therapies for myeloproliferative neoplasms. Biomarker Research.

[27] Byrd JC, Harrington B, O'Brien S, Jones JA, Schuh A, Devereux S, Chaves J, Wierda WG, Awan FT, Brown JR, Hillmen P, Stephens DM, Ghia P, Barrientos JC, Pagel JM, Woyach J, Johnson D, Huang J, Wang X, Kaptein A, Lannutti BJ, Covey T, Fardis M, McGreivy J, Hamdy A, Rothbaum W, Izumi R, Diacovo TG, Johnson AJ, Furman RR (2016). Acalabrutinib (ACP-196) in relapsed chronic lymphocytic leukemia. N Engl J Med.

[28] Perini GF, Ribeiro GN, Pinto Neto JV, Campos LT, Hamerschlak N (2018). BCL-2 as therapeutic target for hematological malignancies. J Hematol Oncol.

[29] Wang ML, Rule S, Martin P, Goy A, Auer R, Kahl BS, Jurczak W, Advani RH, Romaguera JE, Williams ME, Barrientos JC, Chmielowska E, Radford J, Stilgenbauer S, Dreyling M, Jedrzejczak WW, Johnson P, Spurgeon SE, Li L, Zhang L, Newberry K, Ou Z, Cheng N, Fang B, McGreivy J, Clow F, Buggy JJ, Chang BY, Beaupre DM, Kunkel LA (2013). Targeting BTK with ibrutinib in relapsed or refractory mantle-cell lymphoma. N Engl J Med.

[30] Ayyappan S, Maddocks K (2019). Novel and emerging therapies for B cell lymphoma. J Hematol Oncol.

[31] Gu R, Yang X, Wei H (2018). Molecular landscape and targeted therapy of acute myeloid leukemia. Biomarker Research.

[32] Lai C, Doucette K, Norsworthy K (2019). Recent drug approvals for acute myeloid leukemia. J Hematol Oncol.

[33] Ling Y, Xie Q, Zhang Z, Zhang H (2018). Protein kinase inhibitors for acute leukemia. Biomarker Research.

[34] Liu X, Gong Y (2019). Isocitrate dehydrogenase inhibitors in acute myeloid leukemia. Biomarker Research.

[35] Winer ES, Stone RM (2019). Novel therapy in acute myeloid leukemia (AML): moving toward targeted approaches. Therapeutic advances in hematology.

[36] Kwak EL, Bang Y-J, Camidge DR, Shaw AT, Solomon B, Maki RG, Ou S-HI, Dezube BJ, Jänne PA, Costa DB, Varella-Garcia M, Kim W-H, Lynch TJ, Fidias P, Stubbs H, Engelman JA, Sequist LV, Tan W, Gandhi L, Mino-Kenudson M, Wei GC, Shreeve SM, Ratain MJ, Settleman J, Christensen JG, Haber DA, Wilner K, Salgia R, Shapiro GI, Clark JW (2010). Anaplastic lymphoma kinase inhibition in non–small-cell lung Cancer. N Engl J Med.

[37] Loriot Y, Necchi A, Park SH, Garcia-Donas J, Huddart R, Burgess E, Fleming M, Rezazadeh A, Mellado B, Varlamov S, Joshi M, Duran I, Tagawa ST, Zakharia Y, Zhong B, Stuyckens K, Santiago-Walker A, De Porre P, O’Hagan A, Avadhani A, Siefker-Radtke AO (2019). Erdafitinib in locally advanced or metastatic Urothelial carcinoma. N Engl J Med.

[38] Wang Q, Yang S, Wang K, Sun S-Y (2019). MET inhibitors for targeted therapy of EGFR TKI-resistant lung cancer. J Hematol Oncol.

[39] Zarrabi K, Paroya A, Wu S (2019). Emerging therapeutic agents for genitourinary cancers. J Hematol Oncol.

[40] Zhu X-D, Sun H-C (2019). Emerging agents and regimens for hepatocellular carcinoma. J Hematol Oncol.

[41] Hayes DF (2019). HER2 and breast Cancer — a phenomenal success story. N Engl J Med.

[42] Solomon BJ, Mok T, Kim D-W, Wu Y-L, Nakagawa K, Mekhail T, Felip E, Cappuzzo F, Paolini J, Usari T, Iyer S, Reisman A, Wilner KD, Tursi J, Blackhall F (2014). First-line Crizotinib versus chemotherapy in ALK-positive lung Cancer. N Engl J Med.

[43] Flaherty KT, Puzanov I, Kim KB, Ribas A, McArthur GA, Sosman JA, O'Dwyer PJ, Lee RJ, Grippo JF, Nolop K, Chapman PB (2010). Inhibition of mutated, activated BRAF in metastatic melanoma. N Engl J Med.

[44] Long GV, Stroyakovskiy D, Gogas H, Levchenko E, de Braud F, Larkin J, Garbe C, Jouary T, Hauschild A, Grob JJ, Chiarion Sileni V, Lebbe C, Mandalà M, Millward M, Arance A, Bondarenko I, Haanen JBAG, Hansson J, Utikal J, Ferraresi V, Kovalenko N, Mohr P, Probachai V, Schadendorf D, Nathan P, Robert C, Ribas A, DeMarini DJ, Irani JG, Casey M (2014). Combined BRAF and MEK inhibition versus BRAF inhibition alone in melanoma. N Engl J Med.

[45] Coleman RL, Fleming GF, Brady MF, Swisher EM, Steffensen KD, Friedlander M, Okamoto A, Moore KN, Efrat Ben-Baruch N, Werner TL, Cloven NG, Oaknin A, DiSilvestro PA, Morgan MA, Nam J-H, Leath CA, Nicum S, Hagemann AR, Littell RD, Cella D, Baron-Hay S, Garcia-Donas J, Mizuno M, Bell-McGuinn K, Sullivan DM, Bach BA, Bhattacharya S, Ratajczak CK, Ansell PJ, Dinh MH, et al. Veliparib with first-line chemotherapy and as maintenance therapy in ovarian Cancer. N Engl J Med. 2019. 10.1056/NEJMoa1909707 PMC6941439 31562800

[46] Fong PC, Boss DS, Yap TA, Tutt A, Wu P, Mergui-Roelvink M, Mortimer P, Swaisland H, Lau A, O'Connor MJ, Ashworth A, Carmichael J, Kaye SB, Schellens JHM, de Bono JS (2009). Inhibition of poly(ADP-ribose) polymerase in tumors from BRCA mutation carriers. N Engl J Med.

[47] González-Martín A, Pothuri B, Vergote I, DePont Christensen R, Graybill W, Mirza MR, McCormick C, Lorusso D, Hoskins P, Freyer G, Baumann K, Jardon K, Redondo a, Moore RG, Vulsteke C, O’Cearbhaill RE, Lund B, Backes F, Barretina-Ginesta P, Haggerty AF, Rubio-Pérez MJ, Shahin MS, Mangili G, Bradley WH, Bruchim I, Sun K, Malinowska IA, Li Y, Gupta D, Monk BJ: Niraparib in Patients with Newly Diagnosed Advanced Ovarian Cancer. *New England Journal of Medicine* 2019.

[48] Hortobagyi GN, Stemmer SM, Burris HA, Yap Y-S, Sonke GS, Paluch-Shimon S, Campone M, Blackwell KL, André F, Winer EP, Janni W, Verma S, Conte P, Arteaga CL, Cameron DA, Petrakova K, Hart LL, Villanueva C, Chan A, Jakobsen E, Nusch A, Burdaeva O, Grischke E-M, Alba E, Wist E, Marschner N, Favret AM, Yardley D, Bachelot T, Tseng L-M (2016). Ribociclib as first-line therapy for HR-positive, advanced breast Cancer. N Engl J Med.

[49] Turner NC, Ro J, André F, Loi S, Verma S, Iwata H, Harbeck N, Loibl S, Huang Bartlett C, Zhang K, Giorgetti C, Randolph S, Koehler M, Cristofanilli M (2015). Palbociclib in hormone-receptor–positive advanced breast Cancer. N Engl J Med.

[50] Wolff AC (2016). CDK4 and CDK6 inhibition in breast Cancer — a new standard. N Engl J Med.

[51] Wang J, Yuan R, Song W, Sun J, Liu D, Li Z (2017). PD-1, PD-L1 (B7-H1) and tumor-site immune modulation therapy: the historical perspective. J Hematol Oncol.

[52] Wang D, Lin J, Yang X, Long J, Bai Y, Yang X, Mao Y, Sang X, Seery S, Zhao H (2019). Combination regimens with PD-1/PD-L1 immune checkpoint inhibitors for gastrointestinal malignancies. J Hematol Oncol.

[53] Yang S, Zhang Z, Wang Q (2019). Emerging therapies for small cell lung cancer. J Hematol Oncol.

[54] Zhang C, Leighl NB, Wu Y-L, Zhong W-Z (2019). Emerging therapies for non-small cell lung cancer. J Hematol Oncol.

[55] Akinleye A, Rasool Z (2019). Immune checkpoint inhibitors of PD-L1 as cancer therapeutics. J Hematol Oncol.

[56] Belkaid Y, Hand TW (2014). Role of the microbiota in immunity and inflammation. Cell.

[57] Porter DL, Levine BL, Kalos M, Bagg A, June CH (2011). Chimeric antigen receptor-modified T cells in chronic lymphoid leukemia. N Engl J Med.

[58] Raje N, Berdeja J, Lin Y, Siegel D, Jagannath S, Madduri D, Liedtke M, Rosenblatt J, Maus MV, Turka A, Lam L-P, Morgan RA, Friedman K, Massaro M, Wang J, Russotti G, Yang Z, Campbell T, Hege K, Petrocca F, Quigley MT, Munshi N, Kochenderfer JN (2019). Anti-BCMA CAR T-cell therapy bb2121 in relapsed or refractory multiple myeloma. N Engl J Med.

[59] Zhao W-H, Liu J, Wang B-Y, Chen Y-X, Cao X-M, Yang Y, Zhang Y-L, Wang F-X, Zhang P-Y, Lei B, Gu L-F, Wang J-L, Yang N, Zhang R, Zhang H, Shen Y, Bai J, Xu Y, Wang X-G, Zhang R-L, Wei L-L, Li Z-F, Li Z-Z, Geng Y, He Q, Zhuang Q-C, Fan X-H, He A-L, Zhang W-G (2018). A phase 1, open-label study of LCAR-B38M, a chimeric antigen receptor T cell therapy directed against B cell maturation antigen, in patients with relapsed or refractory multiple myeloma. J Hematol Oncol.

[60] Garfall AL, Stadtmauer EA, Hwang WT, Lacey SF, Melenhorst JJ, Krevvata M, Carroll MP, Matsui WH, Wang Q, Dhodapkar MV, Dhodapkar K, Das R, Vogl DT, Weiss BM, Cohen AD, Mangan PA, Ayers EC, Nunez-Cruz S, Kulikovskaya I, Davis MM, Lamontagne A, Dengel K, Kerr ND, Young RM, Siegel DL, Levine BL, Milone MC, Maus MV, June CH (2018). Anti-CD19 CAR T cells with high-dose melphalan and autologous stem cell transplantation for refractory multiple myeloma. JCI Insight.

[61] June CH, O'Connor RS, Kawalekar OU, Ghassemi S, Milone MC (2018). CAR T cell immunotherapy for human cancer. Science.

[62] June CH, Sadelain M (2018). Chimeric antigen receptor therapy. N Engl J Med.

[63] Lv J, Zhao R, Wu D, Zheng D, Wu Z, Shi J, Wei X, Wu Q, Long Y, Lin S, Wang S, Wang Z, Li Y, Chen Y, He Q, Chen S, Yao H, Liu Z, Tang Z, Yao Y, Pei D, Liu P, Zhang X, Zhang Z, Cui S, Chen R, Li P (2019). Mesothelin is a target of chimeric antigen receptor T cells for treating gastric cancer. J Hematol Oncol.

[64] Wei J, Han X, Bo J, Han W (2019). Target selection for CAR-T therapy. J Hematol Oncol.

[65] Ma H, Padmanabhan IS, Parmar S, Gong Y (2019). Targeting CLL-1 for acute myeloid leukemia therapy. J Hematol Oncol.

[66] Mizukoshi E, Kaneko S (2019). Immune cell therapy for hepatocellular carcinoma. J Hematol Oncol.

[67] Jia H, Wang Z, Wang Y, Liu Y, Dai H, Tong C, Guo Y, Guo B, Ti D, Han X, Yang Q, Wu Z, Han W (2019). Haploidentical CD19/CD22 bispecific CAR-T cells induced MRD-negative remission in a patient with relapsed and refractory adult B-ALL after haploidentical hematopoietic stem cell transplantation. J Hematol Oncol.

[68] Zhao J, Lin Q, Song Y, Liu D (2018). Universal CARs, universal T cells, and universal CAR T cells. J Hematol Oncol.

[69] Benjamin R, Graham C, Yallop D, Jozwik A, Ciocarlie O, Jain N, Jabbour EJ, Maus MV, Frigault M, Boissel N, Larghero J, Baruchel A, Mohty M, De Moerloose B, Bloor A, Frey NV, Zinaï A, Balandraud S, Philippe A, Fouliard S, Gauthier L, Pauly J, Konto C, Bermingham C, Veys P, Qasim W: Preliminary Data on Safety, Cellular Kinetics and Anti-Leukemic Activity of UCART19, an Allogeneic Anti-CD19 CAR T-Cell Product, in a Pool of Adult and Pediatric Patients with High-Risk CD19+ Relapsed/Refractory B-Cell Acute Lymphoblastic Leukemia. *Blood* 2018, 132(Suppl 1):896–896.

[70] Giavridis T, van der Stegen SJC, Eyquem J, Hamieh M, Piersigilli A, Sadelain M (2018). CAR T cell-induced cytokine release syndrome is mediated by macrophages and abated by IL-1 blockade. Nat Med.

[71] Norelli M, Camisa B, Barbiera G, Falcone L, Purevdorj A, Genua M, Sanvito F, Ponzoni M, Doglioni C, Cristofori P, Traversari C, Bordignon C, Ciceri F, Ostuni R, Bonini C, Casucci M, Bondanza A (2018). Monocyte-derived IL-1 and IL-6 are differentially required for cytokine-release syndrome and neurotoxicity due to CAR T cells. Nat Med.

[72] Neelapu SS, Tummala S, Kebriaei P, Wierda W, Locke FL, Lin Y, Jain N, Daver N, Gulbis AM, Adkins S, Rezvani K, Hwu P, Shpall EJ (2018). Toxicity management after chimeric antigen receptor T cell therapy: one size does not fit 'ALL'. Nat Rev Clin Oncol.

[73] Porter D, Frey N, Wood PA, Weng Y, Grupp SA (2018). Grading of cytokine release syndrome associated with the CAR T cell therapy tisagenlecleucel. J Hematol Oncol.

[74] Wang Z, Han W (2018). Biomarkers of cytokine release syndrome and neurotoxicity related to CAR-T cell therapy. Biomarker Research.

[75] Hua H, Kong Q, Zhang H, Wang J, Luo T, Jiang Y (2019). Targeting mTOR for cancer therapy. J Hematol Oncol.

[76] Xu S, Li X, Liu Y, Xia Y, Chang R, Zhang C (2019). Inflammasome inhibitors: promising therapeutic approaches against cancer. J Hematol Oncol.

[77] Lin Y, Xu J, Lan H (2019). Tumor-associated macrophages in tumor metastasis: biological roles and clinical therapeutic applications. J Hematol Oncol.

[78] Shang L, Buatois V, Hatterer E, Chauchet X, Haddouk H, Majocchi S, Masternak K, Kosco-Vilbois MH, Fischer N, Ferlin WG (2019). Abstract 546: selectively targeting CD47 with bispecific antibody to efficiently eliminate mesothelin-positive solid tumors. Cancer Res.

[79] Gholamin Sharareh, Mitra Siddhartha S., Feroze Abdullah H., Liu Jie, Kahn Suzana A., Zhang Michael, Esparza Rogelio, Richard Chase, Ramaswamy Vijay, Remke Marc, Volkmer Anne K., Willingham Stephen, Ponnuswami Anitha, McCarty Aaron, Lovelace Patricia, Storm Theresa A., Schubert Simone, Hutter Gregor, Narayanan Cyndhavi, Chu Pauline, Raabe Eric H., Harsh Griffith, Taylor Michael D., Monje Michelle, Cho Yoon-Jae, Majeti Ravi, Volkmer Jens P., Fisher Paul G., Grant Gerald, Steinberg Gary K., Vogel Hannes, Edwards Michael, Weissman Irving L., Cheshier Samuel H. (2017). Disrupting the CD47-SIRPα anti-phagocytic axis by a humanized anti-CD47 antibody is an efficacious treatment for malignant pediatric brain tumors. Science Translational Medicine.

[80] Lawler SE, Speranza M-C, Cho C-F, Chiocca EA (2017). Oncolytic viruses in Cancer treatment: a review. JAMA Oncology.

[81] Li Z, Song W, Rubinstein M, Liu D (2018). Recent updates in cancer immunotherapy: a comprehensive review and perspective of the 2018 China Cancer immunotherapy workshop in Beijing. J Hematol Oncol.

[82] He Q, Liu Z, Liu Z, Lai Y, Zhou X, Weng J (2019). TCR-like antibodies in cancer immunotherapy. J Hematol Oncol.

[83] Ishihara Mikiya, Kitano Shigehisa, Hattori Hiroyoshi, Miyahara Yoshihiro, Kato Hidefumi, Mishima Hideyuki, Yamamoto Noboru, Funakoshi Takeru, Kojima Takashi, Sasada Tetsuro, Sato Eiichi, Okamoto Sachiko, Tomura Daisuke, Chono Hideto, Nukaya Ikuei, Mineno Junichi, Ikeda Hiroaki, Watanabe Takashi, Kageyama Shinichi, Shiku Hiroshi (2019). Tumor responses and early onset cytokine release syndrome in synovial sarcoma patients treated with a novel affinity-enhanced NY-ESO-1-targeting TCR-redirected T cell transfer. Journal of Clinical Oncology.

